# Reigniting hope in cancer treatment: the promise and pitfalls of IL-2 and IL-2R targeting strategies

**DOI:** 10.1186/s12943-023-01826-7

**Published:** 2023-07-29

**Authors:** Shan Muhammad, Tao Fan, Yang Hai, Yibo Gao, Jie He

**Affiliations:** 1 Department of Thoracic Surgery, National Clinical Research Center for Cancer/Cancer Hospital, National Cancer Center, Chinese Academy of Medical Sciences and Peking Union Medical College, Beijing, 100021 China; 2 Present Address: Laboratory of Translational Medicine, National Clinical Research Center for Cancer/Cancer Hospital, National Cancer Center, Chinese Academy of Medical Sciences and Peking Union Medical College, Beijing, 100021 China; 3 Department of Colorectal Surgery, National Clinical Research Center for Cancer/Cancer Hospital, National Cancer Center, Chinese Academy of Medical Sciences & Peking Union Medical College, Beijing, 100021 China; 4 Department of Colorectal Surgery, The Second Affiliated Hospital of Harbin Medical University, Harbin, 150086 China; 5 State Key Laboratory of Molecular Oncology, National Cancer Center/National Clinical Research Center for Cancer/Cancer Hospital, Chinese Academy of Medical Sciences and Peking Union Medical College, Beijing, 100021 China; 6 Department of Children’s and Adolescent Health, Public Health College of Harbin Medical University, 157 Baojian Road, Harbin, 150081 China; 7 Present Address: Central Laboratory & Shenzhen Key Laboratory of Epigenetics and Precision Medicine for Cancers, National Cancer Center/National Clinical Research Center for Cancer/Cancer Hospital & Shenzhen Hospital, Chinese Academy of Medical Sciences and Peking Union Medical College, Shenzhen, 518116 China

**Keywords:** Interleukin-2 (IL-2), IL-2 receptor (IL-2R), Tumor microenvironment, IL-2-based immunotherapy, Regulatory T cells, Cancer biomarkers, Engineered IL-2 variants, Combination cancer therapy, Checkpoint inhibitors, Tumor immune response, Dose optimization of IL-2, Cancer prognosis and IL-2, Immune activation and tolerance, Tumorigenesis, Personalized cancer treatment, Cancer immunosurveillance

## Abstract

Interleukin-2 (IL-2) and its receptor (IL-2R) are essential in orchestrating immune responses. Their function and expression in the tumor microenvironment make them attractive targets for immunotherapy, leading to the development of IL-2/IL-2R-targeted therapeutic strategies. However, the dynamic interplay between IL-2/IL-2R and various immune cells and their dual roles in promoting immune activation and tolerance presents a complex landscape for clinical exploitation. This review discusses the pivotal roles of IL-2 and IL-2R in tumorigenesis, shedding light on their potential as diagnostic and prognostic markers and their therapeutic manipulation in cancer. It underlines the necessity to balance the anti-tumor activity with regulatory T-cell expansion and evaluates strategies such as dose optimization and selective targeting for enhanced therapeutic effectiveness. The article explores recent advancements in the field, including developing genetically engineered IL-2 variants, combining IL-2/IL-2R-targeted therapies with other cancer treatments, and the potential benefits of a multidimensional approach integrating molecular profiling, immunological analyses, and clinical data. The review concludes that a deeper understanding of IL-2/IL-2R interactions within the tumor microenvironment is crucial for realizing the full potential of IL-2-based therapies, heralding the promise of improved outcomes for cancer patients.

**Supplementary Information:**

The online version contains supplementary material available at 10.1186/s12943-023-01826-7.

## Introduction

Interleukin-2 (IL-2) is a cytokine produced by certain immune cells, such as T and B cells, which plays a crucial role in regulating the immune system by stimulating the proliferation and activation of these cells [1]. The Interleukin-2 receptor (IL-2R), a transmembrane glycoprotein receptor, is prominently located on the surface of T and B cells, among other immune system cells. This receptor has a crucial role in the immune response, as it binds to IL-2, instigating a cascade of events that culminate in the activation and proliferation of these cells. This process underscores the fundamental role of IL-2R in the modulation and functioning of the immune system [2]. The cooperative action of IL-2 and IL-2R is necessary for the proper functioning of the immune system, as it helps to maintain the balance between the activation and suppression of the immune response to pathogens [3].

Despite the beneficial role of IL-2 in instigating immune responses to attack tumor cells, its ability to expand regulatory T cells (Tregs), potentially dampening anti-tumor immunity, presents a nuanced and paradoxical situation. The intriguing complexity of the IL-2 and IL-2R signaling pathways extends beyond routine immune regulation, with compelling evidence illuminating their roles within the tumor microenvironment (TME). In addition, IL-2 and IL-2R have also been found to play a significant role in the development and progression of cancer [4]. Moreover, IL-2 has been shown to promote the growth and survival of specific tumor cells, and studies have demonstrated that IL-2R is involved in the angiogenesis or growth of new blood vessels in tumors [5–8]. Furthermore, IL-2 and IL-2R have been implicated in the metastatic process, whereby cancer cells migrate from the primary tumor to distant body parts [8, 9].

Consequently, therapies targeting IL-2 and IL-2R are currently under development and rigorous testing as potential oncological treatments, demonstrating encouraging outcomes in clinical trials [5, 10, 11]. These innovative therapeutic strategies aim to invigorate the body’s immune system, enabling it to identify and subsequently eradicate cancer cells. The pivotal role of IL-2 and IL-2R in this immunological response underscores their significance in the advancement of cancer treatment modalities. IL-2 has been shown to activate T cells, which can then target and kill cancer cells, while IL-2R is expressed on the surface of certain immune cells, such as natural killer (NK) cells, and can help to stimulate these cells to fight cancer [12, 13].

Although IL-2 has been utilized in immunotherapy, the success of these strategies is variably influenced by the differential expression of IL-2 and IL-2R across diverse cancer types. For instance, IL-2-based immunotherapy has demonstrated increased efficacy in malignancies such as melanoma and renal cell carcinoma (RCC), attributed to their higher expression of IL-2R ([7, 8]). In addition, IL-2 and IL-2R are involved in several cancer-related pathways. For example, IL-2 has been shown to regulate cell proliferation, apoptosis, and angiogenesis, while IL-2R has been implicated in tumor growth, metastasis, and immune evasion [5, 14–16]. In targeting IL-2 and IL-2R-mediated pathways, there is a promising approach for treating cancer, and various IL-2 and IL-2R-targeted therapies are currently being developed and tested in clinical trials [12, 17, 18].

However, there are still critical challenges in the therapeutic targeting of IL-2 and IL-2R in cancer, such as the lack of specificity of these molecules and the potential for side effects. For instance, IL-2 is known to regulate several immune pathways, and targeting it could lead to an over-activation of the immune system, resulting in adverse effects [19–22]. Therefore, this review article aims to delineate the dichotomy of IL-2 and IL-2R’s functions within the tumor milieu and its implications for cancer immunotherapy.

Our understanding of the IL-2 and IL-2R pathways within the context of cancer has significantly evolved, opening avenues for improved and safer therapeutic interventions. Nevertheless, there is still much to discern. This review seeks to address the key questions: How can we balance the dual roles of IL-2 to optimize its anti-tumor effects? Furthermore, how do the IL-2 and IL-2R expression variations among different cancer types influence the therapeutic response? Moreover, lastly, what is the potential of IL-2 and IL-2R as diagnostic, prognostic, or predictive markers for cancer? By addressing these questions, we aim to provide a comprehensive overview of IL-2 and IL-2R’s functions in the TME, catalyzing further research toward developing more effective cancer immunotherapies.

## Deciphering IL-2 and IL-2R: biology, signaling, and regulation

### Mechanisms of IL-2 and IL-2R signaling

IL-2 is a critical cytokine essential in the adaptive immune system’s regulation [23]. Predominantly produced by T helper cells and NK cells upon antigen recognition, IL-2 originates from other immune cells, such as dendritic cells [23, 24]. Its primary function is to stimulate the proliferation and differentiation of T and B lymphocytes [23, 24]. IL-2R is constituted by three unique subunits: CD25, also denoted as the alpha chain or IL-2Rα; CD122, identified as the beta chain or IL-2Rβ; and CD132, referred to as the gamma chain or IL-2Rγ [24, 25]. These chains contribute uniquely to the IL-2R’s functionality. IL-2 binding initiates via CD25, enhancing the receptor’s affinity for the cytokine [24, 25]. CD122, predominantly expressed in NK cells, is also found in other immune cells, playing a crucial role in cytokine binding and signal transduction1. CD132 assists in assembling the receptor complex and participates in signaling, contributing to the overall immune response [24, 25].

**Fig. 1:**
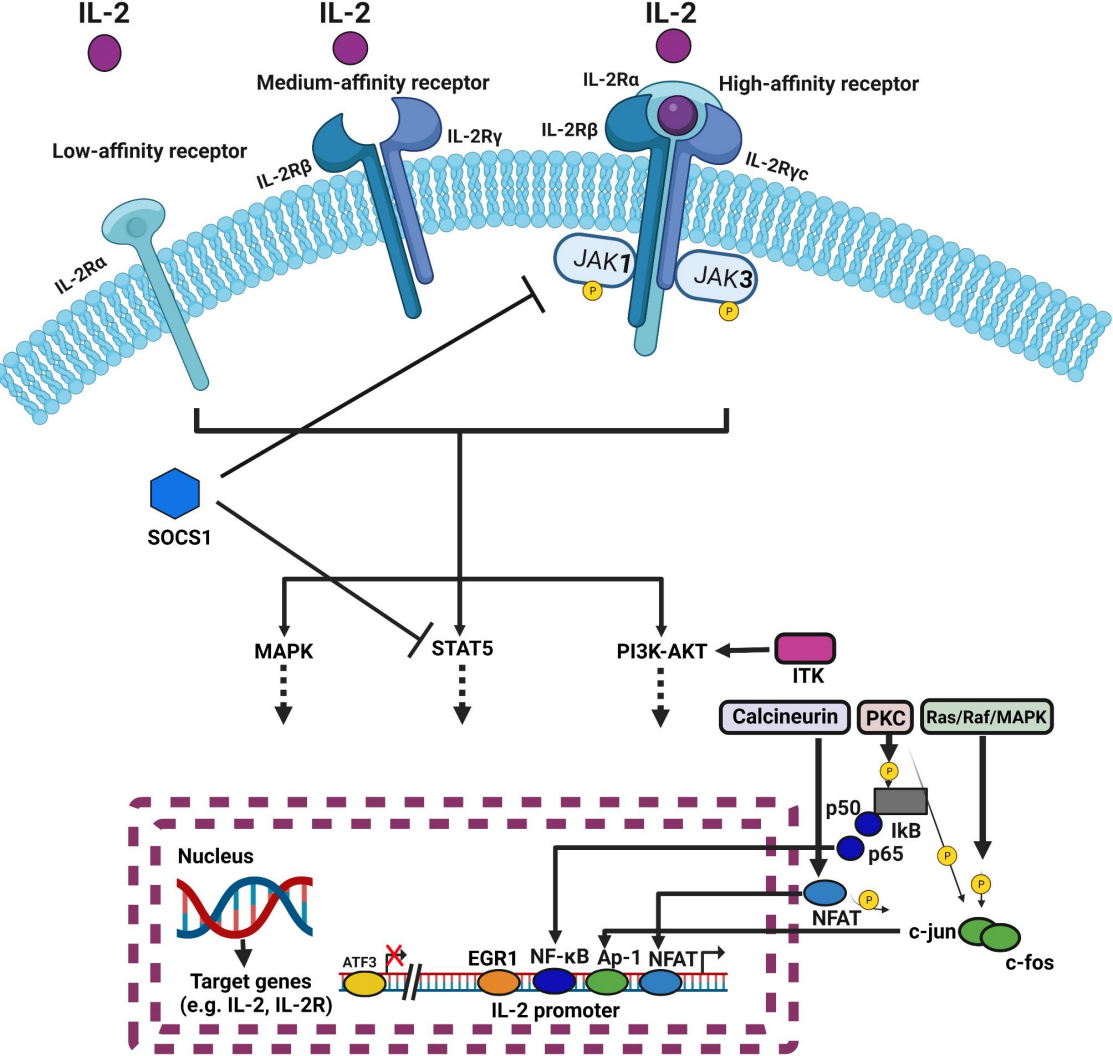
This diagram provides a comprehensive illustration of the interleukin-2 (IL-2) and interleukin-2 receptor (IL-2R) pathway within the tumor microenvironment, detailing its intrinsic role in tumor immunology. IL-2R, encompassing three distinct chains—IL-2Rα (CD25), IL-2Rβ (CD122), and γc (CD132)—comes in three unique forms formed by different combinations of these chains. The depicted schematic demonstrates how the IL-2/IL-2R pathway activates Janus kinase 1 (JAK1) and Janus kinase 3 (JAK3) following their phosphorylation, thereby prompting the downstream signaling across multiple specialized pathways. Each of these pathways displays unique modulation within regulatory T cells (Treg cells), differing distinctly from effector T cells (Teff) or conventional T cells (Tconv). Notable components that demonstrate increased activity within Treg cells, in comparison to Teff or Tconv cells, include the signal transducer and activator of transcription 5 (STAT5) and phosphatase and tensin homolog (PTEN). On the other hand, the phosphatidylinositol 3-kinase (PI3K)/Akt and mitogen-activated protein kinase (MAPK)/extracellular signal–related kinase (Erk) pathways show enhanced activity within Teff or Tconv cells. Additionally, other significant transcription factors and regulators like IL2-inducible T-cell kinase (ITK), inhibitors of nuclear factor-κB (IκB), Nuclear factor of activated T-cells (NFAT), Activator protein-1 (AP-1), the C-JUN protein—a signal-transducing transcription factor of the AP-1 family, and Protein c-Fos—a proto-oncogene that is the human homolog of the retroviral oncogene v-fos, are also engaged in the IL-2 and IL-2R signaling pathway. These components further modulate the intricate signaling dynamics within the Treg, Teff, and Tconv cells. The Suppressors of Cytokine Signaling1 (SOCS1), a critical regulator of cytokine signaling, is also implicated in this pathway and contributes to the complex interplay of signals within the tumor microenvironment. This detailed insight into the IL-2/IL-2R signaling pathway, along with the roles of the associated proteins, aims to facilitate an enhanced understanding of tumor immunology and could possibly pave the way for novel therapeutic strategies. The figure was created using the online tool https://biorender.com/

The IL-2R/JAK/STAT signaling pathway is instrumental in T and B lymphocyte development and functional maturation. Upon activation, this pathway orchestrates the nuclear translocation of specific transcription factors, thereby regulating the expression of multiple target genes, which notably include IL-2 itself. This results in a self-perpetuating positive feedback loop [23, 26]. In addition to the JAK/STAT pathway, IL-2R activation also instigates two other critical signaling cascades: the Mitogen-Activated Protein Kinase (MAPK) pathway [26, 27], and the Phosphatidylinositol 3-Kinase (PI3K) pathway [28]. While the MAPK pathway is crucial for regulating cell proliferation and differentiation processes, the PI3K pathway is instrumental in ensuring cell survival and promoting lymphocyte proliferation and differentiation [28]. (Figure.1).

IL-2, a potent lymphocyte growth factor, utilizes these signaling pathways to govern various aspects of the adaptive immune response. For instance, it fosters the proliferation and differentiation of CD4 + T cells into Th1 and Th2 cells [29, 30], while it aids CD8 + T cells in their development into cytotoxic T lymphocytes (CTLs) [31]. Additionally, IL-2 influences the B cell lineage by promoting differentiation into plasma cells and contributes to the development of Tregs, a subset critical for maintaining peripheral tolerance [32].

**Fig. 2:**
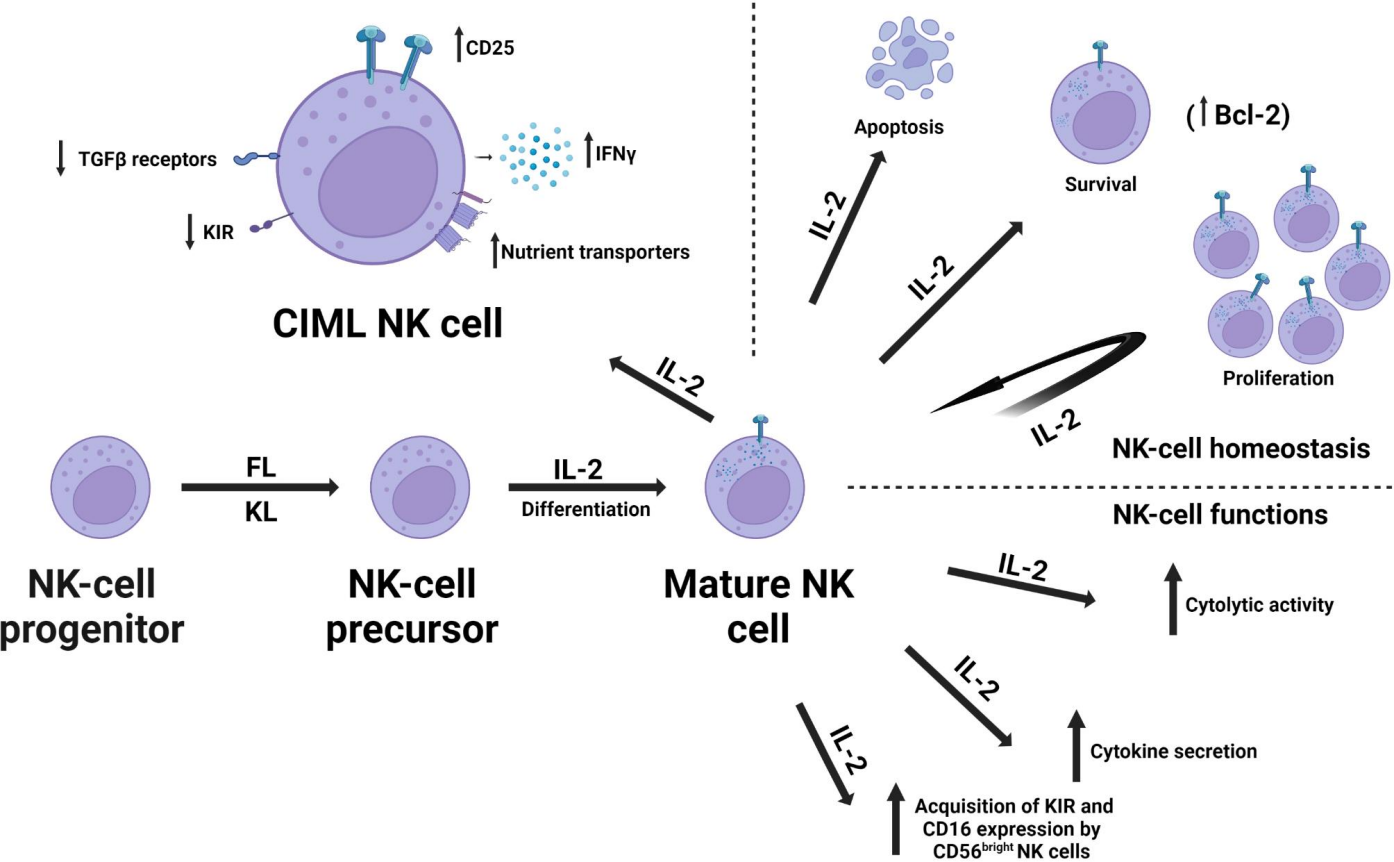
The schematic diagram illustrates the multifaceted role of Interleukin-2 (IL-2) in the homeostasis and functional dynamics of Natural Killer (NK) cells, which are characterized as CD56^+^CD3^−^ large granular lymphocytes integral to the human innate immune response. IL-2 is instrumental in the differentiation process, facilitating the transformation of NK precursors into mature NK cells. It also maintains the homeostasis of mature NK cells by regulating their survival, proliferation, and apoptosis. The diagram further highlights the influence of IL-2 on the functional aspects of mature NK cells. It enhances the cytolytic activity of both CD56^bright^ and CD56^dim^ NK cells and significantly amplifies cytokine secretion, particularly within the CD56^bright^ human NK subset. In secondary lymphoid tissues such as lymph nodes, IL-2 promotes the acquisition of Killer Cell Immunoglobulin-like Receptors (KIR; also known as CD158) and CD16 expression by CD56^bright^ NK cells. The diagram also showcases the characteristics of cytokine-induced memory-like (CIML) NK cells, which include increased expression of CD25 (IL-2Ra), decreased expression of KIRs, and Transforming Growth Factor-beta (TGFβ) receptors. These changes potentially alleviate inhibitory mechanisms in CIML NK cells. The enhanced production of Interferon-gamma (IFNγ) may augment anti-tumor responses. The schematic also highlights the metabolic changes in CIML NK cells. Metabolic alterations, including the roles of glucose transporter and transferrin receptor, are emphasized for their critical contribution to the long-term persistence and recall functions of CIML NK cells. Abbreviations: fms-like tyrosine kinase 3 ligand (FL), kit ligand (KL).The figure was created using the online tool https://biorender.com/

Besides T and B cells, IL-2 also profoundly impacts NK cells. By triggering the JAK/STAT pathway, IL-2 instigates the production of Interferon-gamma (IFN-γ), a cytokine essential for the activation and proliferation of NK cells [33]. Furthermore, IL-2 aids in the differentiation of NK cells into effector and memory phenotypes, further expanding its influence over the immune response [34] (Fig. 2).

### Regulation of IL-2 and IL-2R by transcription factors and regulators

The regulation of IL-2 and IL-2R, crucial in governing the immune response, is modulated by several transcription factors [35–37]. Positive regulators such as the Nuclear Factor of Activated T-cells (NFAT) and Activator Protein-1 (AP-1) bind to the promoter and enhancer regions of IL-2, respectively [35, 36]. JAKs, STAT5, and STAT3 also augment IL-2 receptor signaling [37]. Conversely, negative regulators include Suppressor of Cytokine Signaling-1 (SOCS1), which inhibits JAK/STAT signaling [38], and Forkhead box protein 3 (FoxP3), a transcription factorsuppressing IL-2 production and IL-2 receptor expression in regulatory T cells [39, 40] (Fig. 1; Table 1)([41–65]). Balancing these positive and negative regulators of IL-2 and its receptor is crucial for appropriate immune function. Dysregulation can lead to autoimmune disorders and cancer, emphasizing the importance of understanding the biology, signaling pathways, transcription factors, and regulators of IL-2 and IL-2R [35–40].

**Table 1:** Transcription factors and regulators involved in the regulation of Interleukin-2 and its receptor

Regulator	Type	Function	References
Nuclear Factor of Activated T-cells (NFAT)	Transcription factor	Binds to the promoter region of IL-2 to promote its expression	[41], [42]
Activator Protein-1 (AP-1)	Transcription factor	Binds to the enhancer region of IL-2 to promote its expression	[41], [43, 44]
Nuclear Factor-kappa B (NF-kB]	Transcription factor	Binds to the promoter region of IL-2 to promote its expression	[41], [45]
Early Growth Response Protein 1 (EGR1]	Transcription factor	Binds to the promoter region of IL-2 to promote its expression	[41], [46, 47]
Activating Transcription Factor 3 (ATF3)	Transcription factor	Binds to the promoter region of IL-2 to suppress its expression	[41], [44, 48]
Janus kinases (JAKs)	Positive regulator	Activates Signal Transducer and Activator of Transcription-5 (STAT5) to promote IL-2 receptor signaling	[49, 50]
Signal Transducer and Activator of Transcription-5 (STAT5)	Positive regulator	Phosphorylated by JAKs to promote IL-2 receptor signaling	[49–51]
Suppressor of Cytokine Signaling-1 (SOCS1)	Negative regulator	Inhibits JAK/STAT signaling, leading to reduced IL-2 receptor signaling	[52–54]
Forkhead Box P3 (FoxP3)	Negative regulator	Suppresses IL-2 production and IL-2 receptor expression in regulatory T cells	[55–58]
Interleukin-2 Inducible T-cell Kinase (ITK)	Positive regulator	Phosphorylates downstream effectors to promote IL-2 receptor signaling	[59–62]
Protein Kinase C-theta (PKCθ)	Positive regulator	Phosphorylates downstream effectors to promote IL-2 receptor signaling	[63–65]

## IL-2/IL-2R in the TME

**Fig. 3:**
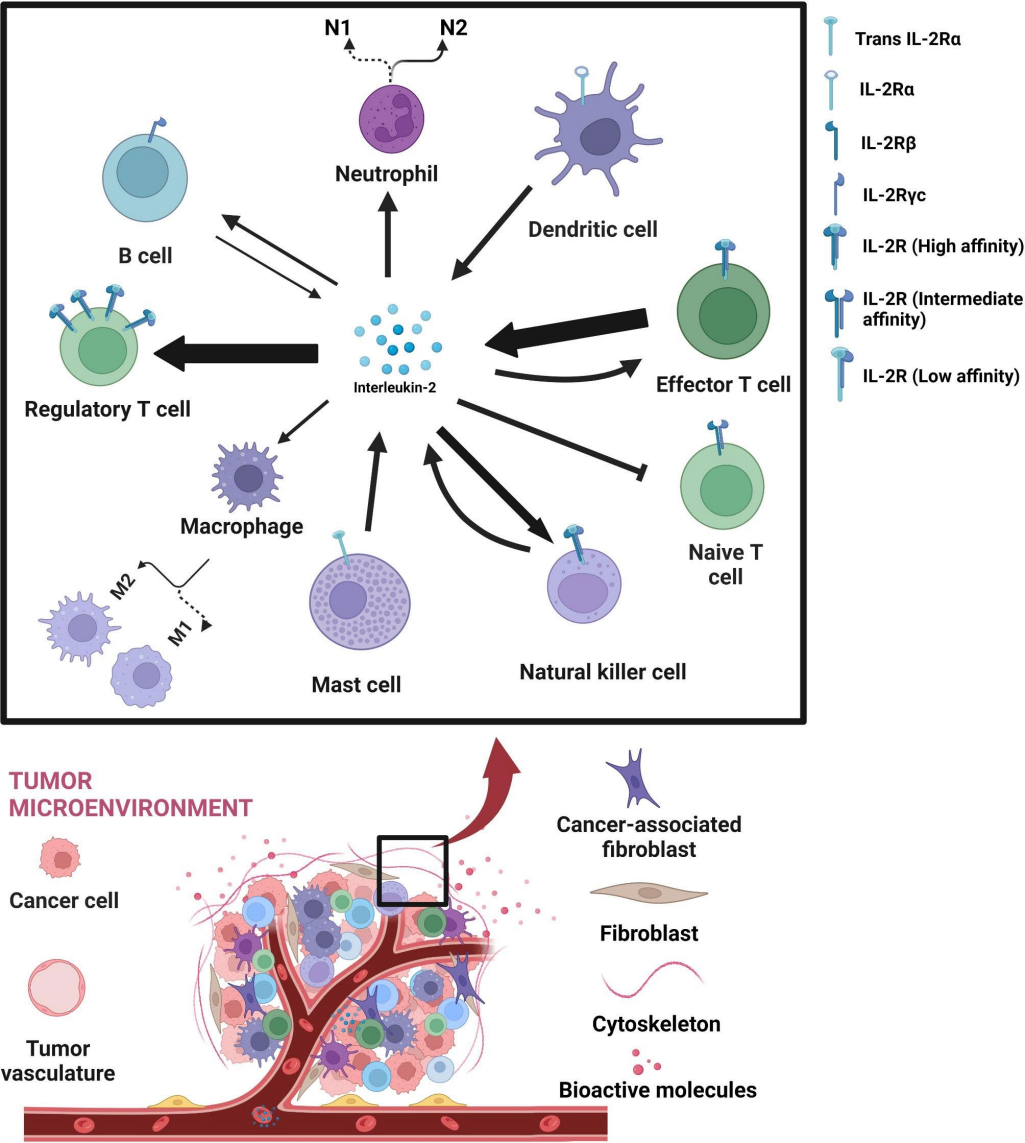
Interplay of IL-2/IL-2R Signaling in the Tumor Microenvironment (TME): This figure elucidates the multifaceted roles of Interleukin-2 (IL-2) and the Interleukin-2 Receptor (IL-2R) within the TME, illustrating the varying influence of IL-2 based on its concentration. Lower levels of IL-2 can foster a regulatory microenvironment which potentially augments tumor growth, while higher levels act as stimulants for immune cells, fostering tumor elimination. Additionally, the figure underscores the heterogeneous impacts of dysregulated IL-2/IL-2R signaling. On one hand, it can stimulate regulatory T cells (Tregs), which may suppress anti-tumor responses, on the other, it can induce infiltration of cytotoxic T cells (CTLs) and NK cells, thus fortifying anti-tumor immune responses. It further emphasizes how IL-2 connects the adaptive and innate immune systems, highlighting the role of cells such as T cells, B cells, NK cells, and dendritic cells, both as IL-2 producers and responders. The varying sizes of arrows in the figure represent the differential intensity of IL-2 production and its effects on different cell types within the TME. Despite its intricate nature, modulating IL-2/IL-2R signaling is underscored as a promising therapeutic avenue, particularly when synergized with other immunotherapies. The figure was created using the online tool https://biorender.com/

TME is a complex, dynamic ecosystem comprising tumor cells, immune cells, stromal cells, and extracellular matrix components that significantly influence tumor growth, invasion, and metastasis [17]. IL-2 and IL-2R have garnered attention within this milieu due to their intricate role in modulating immune responses to tumors [17]. Evidence suggests that dysregulated IL-2/IL-2R signaling within the TME can profoundly impact tumor growth and anti-tumor immune responses. For instance, IL-2/IL-2R signaling may promote the expansion of Tregs, which could potentially inhibit anti-tumor immune responses, thereby facilitating tumor growth [17]. Conversely, other research indicates that IL-2/IL-2R signaling may promote CTL and NK cell infiltration into the TME, enhancing anti-tumor immune responses and impeding tumor growth [66]. It becomes apparent that the role of IL-2/IL-2R signaling in the TME is multifaceted and complex, and a complete understanding of these mechanisms remains a research priority. Promising therapeutic strategies are emerging from these insights. Recent preclinical models demonstrate that IL-2/IL-2R agonists could enhance anti-tumor immune responses and reduce tumor growth [18]. Moreover, combined targeting of IL-2/IL-2R signaling with other immunotherapies may exhibit synergistic effects on tumor growth inhibition [17, 67], (Fig. 3; Table 2) [68–96].

**Table 2:** Approaches and strategies for optimizing IL-2/IL-2R targeted therapies in the context of tumor microenvironment complexity

Approach	Description	Examples	References
Interleukin-2/Interleukin-2 Receptor Axis	The use of IL-2 or agents that target the IL-2 receptor to enhance antitumor immune responses by activating T cells.	High-dose IL-2 therapy, low-dose IL-2 therapy, Treg-depleting antibodies, anti-IL-2 receptor antibodies.	(68, 69, 70, 71)
Combination Therapies	Combination therapies that target multiple components of the tumor micro-environment can enhance treatment efficacy.	Combination of IL-2 with immune checkpoint inhibitors, chemotherapy, radiation therapy, or other immunomodulatory agents.	(10, 72, 73)
Interleukin-2/Interleukin-2 Receptor-Targeted Antibodies	Antibodies that target the IL-2 or IL-2 receptor can enhance antitumor immune responses by blocking inhibitory signals.	Anti-IL-2 antibodies, anti-IL-2 receptor antibodies, anti-CD25 antibodies.	(68, 69, 71, 74, 75)
Small Molecule Inhibitors	Small molecule inhibitors can block signaling pathways that inhibit T cell activation and proliferation in the tumor micro-environment.	JAK inhibitors, MEK inhibitors, PI3K inhibitors.	(76, 77, 78, 79)
Adaptive Dosing	This approach involves adjusting the dose of IL-2 or other immunomodulatory agents based on patient response, with the goal of maximizing treatment efficacy while minimizing toxicity.	Dose escalation or de-escalation of IL-2 based on clinical response, or personalized dosing based on pharmacokinetic and pharmacodynamic parameters.	(80, 81, 82)
Localized Delivery	Localized delivery of IL-2 or other immunomodulatory agents to the tumor micro-environment can enhance treatment efficacy while reducing systemic toxicity.	Localized delivery using drug-eluting implants, nanoparticles, or viral vectors.	(83, 84, 85, 86, 87)
Gene Therapy	Gene therapy involves modifying immune cells or tumor cells to enhance antitumor immune responses. For example, IL-2 gene therapy can be used to deliver IL-2 directly to tumor cells, which may enhance T cell activation and proliferation in the tumor micro-environment.	CAR T cell therapy, tumor-infiltrating lymphocyte (TIL) therapy, or gene therapy using viral vectors to deliver IL-2 or other immunomodulatory agents.	(88, 89, 90, 91, 92)
Combination with Conventional Therapies	IL-2-based therapies can be combined with conventional cancer treatments, such as chemotherapy or radiation therapy, to enhance treatment efficacy.	Combination of IL-2 with cisplatin or vinblastine, or the combination of IL-2 with radiation therapy.	(68, 74, 93, 94, 95, 96)

### IL-2/IL-2R: T-cell regulation within the TME

Within the TME, T-cell function is a critical determinant of tumor growth and progression [97, 98]. Dysregulation of T-cell function, including impaired T-cell receptor signaling, reduced expression of co-stimulatory molecules, and upregulated expression of inhibitory receptors (such as PD-1, CTLA-4, and TIM-3), is a common immune evasion tactic employed by tumors [99–104]. Furthermore, tumors can induce the accumulation and differentiation of Tregs in the TME through the secretion of various cytokines, chemokines, and growth factors [100].

IL-2/IL-2R signaling, an essential player in T-cell regulation, could hold therapeutic potential in rebalancing the TME. Activated T-cells produce IL-2, a cytokine crucial for the proliferation, survival, and function of effector T-cells [29, 105]. IL-2R, expressed on various immune cells, mediates IL-2 signaling and is pivotal in determining the balance between effector T-cells and Tregs within the TME [29, 105]. Specifically, IL-2 signaling can bolster the proliferation and function of effector T-cells while restraining Treg proliferation and function [106]. In addition, IL-2 signaling also promotes the reprogramming of Tregs into effector T-cells, shifting the TME from a pro-tumor to an anti-tumor state [107, 108] (Fig. 3).

Evidence links dysregulated IL-2/IL-2R signaling to poor prognosis in various types of cancer, including melanoma, RCC, and breast cancer [109–112]. Emerging IL-2-based therapies show promise in enhancing the anti-tumor immune response and improving the efficacy of immunotherapy in cancer patients [113].

### IL-2/IL-2R regulatory roles on NK cells, macrophages, neutrophils, and B cells in the TME

The TME is a dynamic network that integrates various cells, including immune and cancer cells, which are instrumental in cancer progression and metastasis [114]. IL-2/IL-2R signaling plays a pivotal role in modulating immune cells, and its dysregulation can contribute to tumor evasion [115]. The following sections delve into the function of this pathway on specific immune cell types in the TME.

### Regulation of NK cells by IL-2/IL-2R

NK cells possess an innate capability to recognize and eliminate malignant cells, and their function is critically modulated by IL-2 [13] (Fig. 2). Zhang et al.‘s study illustrated that IL-2 signaling amplifies the cytotoxic potential of NK cells, thereby inhibiting tumor growth and metastasis. Notably, NK cells recognize and eliminate tumor cells in an MHC-independent manner, distinguishing them from T-cell mediated tumor recognition [116]. Furthermore, this research found that IL-2 treatment in a mouse model of breast cancer reduced tumor growth and metastasis, underscoring the potential for NK cell-based immunotherapies [33, 116].

### IL-2/IL-2R influence on macrophages and neutrophils

Similarly, IL-2 impacts macrophages’ functionality, promoting their differentiation into the M1 phenotype, known for their anti-tumor and potent tumoricidal activities [117–124]. Furthermore, IL-2/IL-2R signaling plays a critical role in neutrophil activity within the TME, modulating their recruitment, activation, and lifespan, thereby influencing tumor progression [118]. A deeper understanding of these mechanisms can pave the way for novel strategies targeting IL-2/IL-2R signaling to enhance neutrophils’ anti-tumor activities or minimize their pro-tumor activities [119–127].

### The role of IL-2/IL-2R signaling in B cells

In comparison to other immune cells, B cells are often overlooked in the TME context. However, recent research has demonstrated the importance of IL-2/IL-2R signaling in regulating B cells within the TME [128]. IL-2 can directly stimulate B cells, enhancing their proliferation, differentiation into antibody-secreting plasma cells, and co-stimulatory molecule expression, such as CD80 and CD86 [128].

However, IL-2/IL-2R signaling can also promote Treg expansion, potentially inhibiting the anti-tumor immune response [129]. The interplay between Tregs, B-cells, and other cytokines and immune cells within the TME adds to the complexity of the immune response [130–137]. The role of IL-2/IL-2R signaling in B-cell responses within the TME requires further exploration to fully understand these mechanisms and develop targeted immunotherapeutic strategies [138].

### Interactions and roles of IL-2/IL-2R in dendritic cells (DCs) and myeloid-derived suppressor cells (MDSCs) within the TME

Critical to regulating anti-tumor immune responses within the TME are immune cells, such as DCs and MDSCs, which are influenced by IL-2 /IL-2R signaling [139–142]. DCs, as professional antigen-presenting cells, initiate and regulate immune responses. IL-2 is pivotal in augmenting their maturation and activation, enhancing antigen presentation capacity, and increasing T-cell activation [139] By upregulating co-stimulatory molecules, such as CD80 and CD86, IL-2 facilitates the activation of naïve T cells [140]. Additionally, IL-2 encourages DC migration to lymph nodes, fostering T cell activation and instigating anti-tumor immune responses [143].

Contrastingly, MDSCs, a diverse population of immature myeloid cells, accumulate within the TME and suppress the anti-tumor immune response. IL-2 exerts a dual role on MDSCs; it stimulates the expansion and accumulation of MDSCs, promoting an immunosuppressive microenvironment [141] while concurrently inducing MDSC differentiation into mature myeloid cells. This differentiation reduces suppressive functionality and potentially fosters anti-tumor immune responses [142]. The IL-2/IL-2R signaling pathway is central to the interplay between DCs and MDSCs within the TME. IL-2, by enhancing the maturation and activation of DCs, promotes antigen presentation and T-cell activation. Consequently, these activated T cells produce IL-2, which induces MDSC differentiation into mature myeloid cells, decreasing immunosuppression within the TME [29]. Nevertheless, IL-2 can simultaneously drive MDSC expansion and accumulation within the TME, creating an immunosuppressive environment [144]. In addition, MDSCs express high levels of IL-2Rα or CD25 [145], implying a possible role for IL-2 signaling in their functionality. Indeed, studies suggest that IL-2 promotes MDSC expansion and activation [29], while blockade of the IL-2/IL-2R pathway may reduce their suppressive function within the TME [144]. Recent studies highlight the intricate relationship between DCs and MDSCs in the TME, regulated by the IL-2/IL-2R pathway. Specifically, IL-2 produced by activated T cells can stimulate MDSC proliferation and differentiation, which may inhibit the maturation and antigen-presenting function of DCs [146, 147]. This reciprocal regulation is thought to contribute to the TME’s immunosuppressive nature, where MDSCs hinder the activation and proliferation of effector T cells. At the same time, DCs struggle to initiate a robust immune response against cancer cells [148].

Contemporary research has probed the role of IL-2 signaling in MDSC differentiation and functionality [149]. It was found that IL-2 signaling is critical for MDSC differentiation and promotion of their immunosuppressive function. This signaling also activates the STAT5 pathway, driving the upregulation of crucial genes involved in MDSC differentiation and functionality [150]. Moreover, IL-2 signaling blockade enhanced the anti-tumor immune response and inhibited tumor growth in murine models [74]. Another study investigated the DC-MDSC interplay within the TME, focusing on the IL-2/IL-2R pathway [151]. They discovered that MDSCs could directly inhibit the function of DCs via the IL-2/IL-2R pathway. Intriguingly, MDSCs express high levels of IL-2R, which competes with DCs for IL-2 binding. This competitive interaction reduces IL-2 signaling in DCs, impairing their function and inhibiting the anti-tumor immune response. However, blocking IL-2R signaling in MDSCs was observed to reverse this effect and enhance DC function [151].

## IL-2 and IL-2R in Cancer Therapy: a balance between immunostimulation and immunosuppression

IL-2 and IL-2R play pivotal roles in the immunological response against cancer, wielding influence over immune regulation and, thus, cancer therapy. Their function extends to the activation of immune cells, such as T cells, B cells, and NK cells, augmenting immune responses against cancer cells. However, their role is dualistic, wielding effects of both immunostimulation and immunosuppression. This duality can present both benefits and challenges in cancer therapy [152–154].

### Immunostimulatory effects of IL-2/IL-2R

IL-2 is instrumental in stimulating the proliferation and activation of T cells and NK cells. This activity enhances the anti-tumor immune response, mainly by IL-2 binding to the high-affinity IL-2R (IL-2Rα/β/γ) expressed on activated T cells [152]. This interaction results in the expansion and activation of effector T cells, responsible for the direct or indirect killing of cancer cells, primarily through cytokine production such as Interferon gamma (IFN-γ) and tumor necrosis factor alpha (TNF-α) [153]. In addition, IL-2 can further induce the expansion of NK cells, which increases tumor-specific cytotoxicity [154]. The efficacy of IL-2 as a cancer immunotherapy has been evaluated in various clinical trials. High-dose IL-2 demonstrates potential, inducing objective responses in up to 15% of patients with metastatic melanoma and RCC, with some patients achieving durable complete responses [7, 10, 91, 155, 156].

### Immunosuppressive role of IL-2/IL-2R

IL-2/IL-2R signaling plays a pivotal role in the activation and proliferation of TILs, immune cells that infiltrate tumors and mediate anti-tumor immune responses. However, the influence of IL-2 on TILs is multifaceted and largely determined by the TME, the presence of Tregs, and the activation state of TILs. These elements can promote the expansion of Tregs and MDSCs that suppress effector T cell activity, thereby paradoxically fostering tumor growth and creating an immunosuppressive effect. Despite the potential of IL-2, the use of high-dose IL-2 is limited due to severe associated toxicities, such as hypotension, pulmonary edema, and renal failure, which restrict its broader clinical use [157–162].

## Strategies for IL-2/IL-2R therapeutic targeting

The intricacies of IL-2 and IL-2R signaling necessitate a delicate balancing act in their therapeutic targeting. Key to this balance is the differential expression of IL-2R subunits on various immune cells [5]. The ratio of IL-2R subunit expression on different cell types can influence the overall effects of IL-2 signaling within the immune system. For instance, targeting IL-2R selectively on effector T cells could enhance the anti-tumor immune response [163]. Conversely, targeting IL-2R on Tregs could lead to an undesirable promotion of immunosuppression and tumor growth [164]. Therefore, IL-2 has been used in combination with other immunotherapies, such as checkpoint inhibitors, to optimize anti-tumor immune responses. For example, high-dose IL-2, combined with ipilimumab (an anti-CTLA-4 antibody), has shown promise by inducing durable responses in patients with metastatic melanoma [165]. However, combination immunotherapy could lead to an escalation in toxicity and the induction of autoimmune reactions.

Consequently, the most practical combination of immunotherapies requires meticulous evaluation in clinical trials. Another promising avenue is using IL-2 as an adjuvant in cancer vaccines to bolster the activation and proliferation of tumor-specific T cells [166–168]. The GVAX vaccine, which consists of irradiated tumor cells genetically modified to secrete GM-CSF, is one such example that has been paired with IL-2 to enhance the anti-tumor immune response [169–171]. Although preclinical models have shown promise, the efficacy of IL-2-based cancer vaccines remains to be verified in clinical trials.

### Challenges in therapeutic targeting of IL-2 and IL-2R in cancer

Despite their immense potential, therapeutic targeting of IL-2 and IL-2R in cancer poses several challenges. A significant challenge is the complexity of IL-2 signaling pathways. IL-2 can activate multiple signaling pathways, including the JAK-STAT, PI3K-AKT, and MAPK pathways, exerting pro- and anti-tumor effects [172, 173]. In addition, the heterogeneity of IL-2R expression on different immune cell populations and the severe side effects associated with IL-2 treatment, such as life-threatening cytokine release syndrome, further complicates the development of effective and safe treatments [174]. Moreover, the issues of delivery and dosing present additional challenges for IL-2-based immunotherapies. As a large protein, IL-2 requires intravenous administration and exhibits a short half-life in vivo, making it challenging to achieve sustained therapeutic levels [13]. In addition, high-dose IL-2, although linked to higher response rates in metastatic melanoma, is associated with increased toxicity [175]. Furthermore, the efficacy of IL-2 and IL-2R-targeted therapies is limited in particular cancer types, such as breast or lung cancer [176].

## Soluble IL-2R (sIL-2R) and IL-2Rα: emerging biomarkers and therapeutic targets in cancer

The IL-2R complex, composed of IL-2Rα, IL-2Rβ, and IL-2Rγ, plays critical roles in the immune system [177, 178]. Each subunit has specific locations and functions within the immune system (Fig. 1; Table 3) [179–198]. Importantly, IL-2Rα, primarily expressed in activated T cells, regulatory T cells, and activated B cells, initiates downstream signaling cascades upon binding to IL-2 [23, 199]. The soluble form of the receptor, sIL-2R, produced by proteolytic cleavage of the membrane-bound IL-2R complex, has been identified in the serum of patients with Hodgkin’s lymphoma and some non-lymphoid cancers [182, 183, 185]. In contrast to its membrane-bound counterpart IL-2Rα, this form presents a longer half-life and different modes of interaction with IL-2 [186, 187]. Both sIL-2R and IL-2Rα have been implicated in numerous immune-mediated diseases, signifying their potential as markers of disease activity and progression [186, 188, 200].

**Table 3:** Summarizing the different sub-units of the Interleukin-2 Receptor, their location and Significance

IL-2 receptor subunit	Location	Function	Soluble form	Type of cancer or cell type	Additional information	Reference
IL-2Rα (CD25)	T cells, B cells, NK cells, monocytes, dendritic cells, endothelial cells	High-affinity binding of IL-2	sIL-2Rα	Various cancers (e.g. leukemia, lymphoma, melanoma, breast, lung, bladder, ovarian, and gastric cancer)	Elevated levels of sIL-2Rα have been detected in the serum of patients with these cancers, and the levels have been shown to correlate with tumor burden, disease stage, and prognosis.	(179, 180, 181, 182, 183, 184, 185, 186, 187, 188, 189, 190)
IL-2Rβ (CD122)	T cells, NK cells, monocytes, dendritic cells	Signal transduction	Not present	Various cancers (e.g. leukemia, lymphoma, melanoma)	Expression of IL-2Rβ has been reported in various cancer types, but its role in cancer development and progression is not well understood.	(10, 190, 191, 192, 193, 194, 195)
IL-2Rγ (CD132)	T cells, NK cells, B cells, monocytes, dendritic cells	Signal transduction	Not present	X-linked severe combined immunodeficiency (XSCID)	Mutations in the IL-2Rγ gene cause XSCID, a severe immunodeficiency disorder that affects T and NK cell function.	(113, 181, 186, 190, 196, 197, 198)

### sIL-2R and IL-2Rα as cancer biomarkers

sIL-2R and IL-2Rα have emerged as promising biomarkers for cancer diagnosis and prognosis. They are found on the surface of cells stimulated by IL-2, with IL-2Rα being a low-affinity receptor whose expression is upregulated in malignant cells [181, 201–203]. Increased levels of sIL-2R and IL-2Rα are observed in several cancers, including colorectal, prostate, breast, and lung cancer [204–207], often indicating a poor prognosis. Moreover, they serve as potential diagnostic markers as elevated levels of sIL-2R and IL-2Rα have been detected in patients with colorectal cancer and those at increased risk of prostate cancer [189, 208]. Further, these markers have potential utility in monitoring treatment efficacy. For example, decreased levels of sIL-2R and IL-2Rα in response to chemotherapy and radiation therapy signify a favorable prognosis in patients with prostate and colorectal cancers [204, 209–211].

### Therapeutic potential of sIL-2R and IL-2Rα

Beyond their roles as biomarkers, sIL-2R and IL-2Rα are under investigation as therapeutic targets for cancer [184, 186, 209, 212, 213]. The ability of sIL-2R to modulate the immune system and tumor microenvironment underlines its potential as cancer therapeutics [10, 188]. It has been proposed that sIL-2R may induce tumor cell apoptosis, inhibit tumor angiogenesis, and interfere with oncogenic pathways [214]. Similar anticancer effects have been attributed to IL-2Rα, including the induction of apoptosis in multiple myeloma cells and the inhibition of oncogenic pathways, such as the JAK-STAT pathway [215–218].

Preliminary clinical studies underscore the therapeutic potential of sIL-2R and IL-2Rα. A phase I trial demonstrated the safety and tolerability of sIL-2R in patients with advanced solid tumors and lymphomas [219–221]. A subsequent phase I/II trial reported a significant reduction in tumor burden in patients with advanced metastatic RCC treated with sIL-2R [222]. Moreover, a phase II trial revealed a significant survival benefit in patients with advanced non-small cell lung cancer (NSCLC) following IL-2Rα treatment [223].

## IL-2 and engineered IL-2 for immunotherapy of autoimmunity and cancer

### IL-2 and engineered IL-2 for immunotherapy of autoimmune disorders

IL-2, a critical cytokine, promotes the growth and activation of T cells, especially Tregs [153]. Tregs are indispensable in controlling immune responses and preventing autoimmune diseases [153]. However, systemic administration of IL-2 is associated with adverse effects due to its pleiotropic effects on various immune cells [71]. A novel strategy developed to mitigate this problem involves receptor-gated IL-2 delivery through an anti-human IL-2 antibody [71]. The receptor-gated IL-2 delivery is a targeted approach where the engineered anti-human IL-2 antibody binds to IL-2 receptors on Tregs, effectively delivering IL-2 specifically to these cells [71]. This enhances Treg activation and their suppressive activity, selectively impacting these cells without disturbing other immune cells [71]. This approach has demonstrated potential in activating Tregs across various species, such as mice, monkeys, and humans [224]. Studies using mouse models of autoimmune diseases like type 1 diabetes, multiple sclerosis, and graft-versus-host disease have illustrated decreased disease severity and improved survival with this approach [71]. Recent studies have also shown that IL-2 therapy can be effective in treating systemic lupus erythematosus by expanding Tregs and reducing disease activity [225].

Recognizing the therapeutic potential of IL-2, researchers are focused on engineering IL-2 for improved efficacy and safety in treating cancer and autoimmune diseases. Modifying IL-2 structure and function can increase its therapeutic potential [226]. Several strategies include designing IL-2 variants to selectively target Tregs [227], modifying the IL-2 receptor to increase Treg selectivity, or creating IL-2 variants with reduced binding affinity to non-specific immune cells [109, 228]. Among the engineered IL-2 variants, “aldesleukin” or “recombinant human IL-2” (rIL-2) has been extensively researched. It has shown promise in enhancing Treg activity and suppressing autoimmune responses, although associated with potential toxicities like vascular leak syndrome (VLS), limiting its clinical application [109, 226, 229, 230].

### IL-2 and engineered IL-2 for cancer immunotherapy

The therapeutic potential of IL-2 has also been recognized in the field of cancer immunotherapy. IL-2 can enhance the anti-tumor immune response and reduce tumor growth, as demonstrated in mouse cancer models [231]. However, the systemic administration of IL-2 is associated with adverse effects, limiting its clinical application [71]. To overcome these limitations, researchers are engineering IL-2 for improved efficacy and safety in cancer treatment.

Further advancements in IL-2 engineering introduced IL-2 “superkines“(discussed beloe), such as “NARA1”. These molecules exhibit enhanced IL-2 receptor binding and improved signaling properties, providing increased potency and selectivity for Tregs compared to rIL-2 [179, 232–234]. However, these superkines may also have the potential to induce autoimmune responses due to their increased activity [234].

Several clinical trials have evaluated the safety and efficacy of these engineered IL-2 molecules for cancer and autoimmune disorders. For example, despite its limitations, Aldesleukin has been FDA-approved for treating metastatic melanoma and RCC [153, 235, 236]. Other engineered IL-2 molecules, like NKTR-214 and AMG 592, have demonstrated selectivity for activating Tregs and NK cells, showing potential in enhancing immune responses against tumors, and are under clinical evaluation [68, 237, 238]. ALT-803, another engineered IL-2 variant, is under clinical trial for treating various cancers, showing promise in preclinical models of multiple myeloma [ClinicalTrials.gov Identifier: NCT02099539]. Moreover, the IL-2 variant ‘tebentafusp’ has shown promise in clinical trials for treating uveal melanoma [239]. It selectively binds to T cells that recognize a specific tumor antigen, leading to their activation and expansion. However, due to its activity on non-tumor-specific T cells, it may also induce autoimmune responses.

Recent studies have also shown that engineered IL-2 molecules can enhance the efficacy of immune checkpoint inhibitors in cancer treatment by promoting the activation and expansion of tumor-specific T cells [240].

While these engineered IL-2 molecules show promise, they also present significant challenges. Potential toxicities, including VLS, can limit their clinical use [109, 239, 241–243]. Other limitations include the potential for inducing autoimmune responses [243], their high cost, and complex manufacturing processes [243, 244].

The burgeoning field of engineered IL-2 for cancer immunotherapy, though in its infancy, carries immense potential, yet it is not devoid of substantial hurdles [243]. A key obstacle lies in the creation of IL-2 variants that can selectively stimulate Tregs without triggering other immune cells [225, 240, 245]. Furthermore, fine-tuning the dosage and administration of engineered IL-2 to strike a balance between therapeutic effectiveness and toxicity presents another significant challenge [243, 246, 247].

Current efforts are directed towards designing IL-2 therapeutics with enhanced in vivo half-lives, targeting specific IL-2 receptor conformations to stimulate specific T cell subsets, or delivering localized therapies to target tissues [241]. A recent trend in the engineering of IL-2 for the therapy of cancer and autoimmunity is the development of PD-1-IL-2R agonists. Deak and colleagues (2022) effectively highlighted the potential of PD-1-IL-2R agonists in eliciting robust and selective immune responses by promoting the expansion of effector T cells without the simultaneous expansion of Tregs. They also underscored the synergistic benefits of PD-1-IL-2R agonists with conventional anti-PD-1 therapy, representing a novel, promising avenue for cancer immunotherapy [248]. Moreover, the study by Zhang et al. (2021) heralded the creation of orthogonal IL-2 systems, serving as a powerful platform for delineating the complex network of cytokine-receptor interactions and their role in immune modulation. By developing murine and human IL-2 orthogonal systems, they demonstrated a controlled activation of IL-2-dependent signaling pathways, providing insights into potential therapeutic strategies for both autoimmune diseases and cancer [249]. Furthermore, Evans et al.‘s work in 1999 on IL-2R-based chimeric molecules paved the way for a better understanding of how IL-2 and its receptor interact, contributing significantly to the development of more effective therapies [250]. These chimeric molecules can harness the power of the immune system more selectively and potently, addressing issues such as toxicity and lack of specificity seen with the conventional IL-2 therapy.

The primary goal of these advancements is to augment therapeutic efficacy while minimizing associated toxicity. Despite these hurdles, engineered IL-2 therapies are poised to become a significant addition to our current arsenal of cancer treatments [241, 251], provided the obstacles of optimizing effectiveness and minimizing potential drawbacks are carefully navigated.

## Future outlooks and perspectives on IL-2-based therapies for immunotherapy: balancing promise and challenges

IL-2, a cytokine critical in T-cell activation and proliferation, serves as a vital component in cancer immunotherapy [252]. While FDA-approved for metastatic RCC and metastatic melanoma, its clinical application remains limited due to associated toxicity [252, 253]. This section delves into recent advancements and future directions in improving the efficacy of IL-2 therapy. The effectiveness of IL-2 therapy can be enhanced by implementing combination therapies. For instance, a phase I trial involving low-dose IL-2 and anti-PD-1 antibody nivolumab in patients with advanced solid tumors yielded a 30% overall response rate [253]. Notably, the toxicity experienced was manageable. Similarly, a combination of IL-2 and a cancer vaccine was evaluated in a phase I trial for patients with metastatic melanoma, which produced a response rate of 44% without significant toxicity [91].

As we continue to delve deeper into the molecular mechanisms and the vast cellular influences of IL-2, our understanding of its therapeutic potential in treating autoimmune diseases and cancer continues to expand. This section presents a comprehensive overview of future outlooks and perspectives, anchored in our current understanding of IL-2 and its applications in immunotherapy.

The regulation of IL-2 and IL-2R, being crucial in governing the immune response, is modulated by several transcription factors [35–37]. Hence, balancing these positive and negative regulators of IL-2 and its receptor is crucial for appropriate immune function. Subsequently, it follows that dysregulation can lead to autoimmune disorders and cancer, emphasizing the importance of understanding the biology, signaling pathways, transcription factors, and regulators of IL-2 and IL-2R [35–40]. In this regard, the IL-2/IL-2R signaling axis, with its profound impact on T-cell function and the overall immune response within the TME, represents a promising target for therapeutic intervention. In this context, it stands to reason that by strategically modulating this signaling pathway, there is potential to enhance the anti-tumor immune response and curtail tumor growth. Given this, understanding and manipulating the role of IL-2 and IL-2R in the regulation of T-cells within the TME may provide potent tools for bolstering the body’s natural defenses against cancer. Furthermore, IL-2 and IL-2R also play regulatory roles on various immune cells, including NK Cells, macrophages, neutrophils, and B Cells in the TME. They also interact significantly with DCs and MDSCs within the TME. Thus, it follows that future investigations should continue to probe the intricate roles and relationships of IL-2/IL-2R, with immune cells, DCs, and MDSCs in the TME. It is conceivable that this exploration may bolster the development of cancer immunotherapies. However, these strategies hinge on a complex and context-dependent relationship between IL-2/IL-2R signaling, DCs, and MDSCs in the TME. Hence, achieving a more profound understanding of this interplay and the mechanisms underpinning the effects of IL-2/IL-2R signaling on DCs and MDSCs is essential [148].

Notably, there is therapeutic potential in sIL-2R and IL-2Rα. Future advancements in cancer therapeutics involving these proteins are expected to encompass the development of novel formulations with improved efficacy and safety. Additionally, ongoing research efforts aim to identify reliable biomarkers of response to sIL-2R and IL-2Rα therapies [14, 23, 177–198, 200]. It is anticipated that such biomarkers could help predict which patients would benefit most from these therapies [201–223, 254, 255]. The burgeoning understanding of IL-2’s molecular mechanisms and cellular influences not only enhances its therapeutic potential in treating autoimmune diseases and cancer but also sets a new paradigm in immunotherapy [68, 71, 153, 226–228]. However, the challenge lies in fine-tuning the properties of engineered IL-2 to stimulate targeted immune responses without provoking adverse effects [109, 225, 240, 243, 245]. Accordingly, key developments encompass the engineering of IL-2 molecules that selectively bind to high-affinity IL-2 receptors on effector T cells and avoid low-affinity receptors on Tregs [68, 241].

In this regard, techniques involving receptor-targeted delivery systems, like anti-human IL-2 antibodies, mark a promising strategy in improving therapeutic potential and reducing systemic administration drawbacks [71, 224]. In turn, the evolution of IL-2 “superkines,“(discussed below) such as “NARA1,“ represents an achievement in IL-2 engineering, featuring enhanced IL-2 receptor binding and signaling properties [179, 232–234]. These innovative strategies aim to enhance therapeutic efficacy while minimizing toxicity, thus underscoring the potential of IL-2-based immunotherapies. However, potential autoimmune responses due to increased activity highlight the need for further investigation [234]. Significantly, the clinical success of engineered IL-2 molecules such as Aldesleukin, despite its limitations, reinforces the potential of IL-2 based therapies [153, 235, 236]. Other IL-2 molecules like NKTR-214 and AMG 592 show promise in preclinical trials, yet challenges of potential toxicities, manufacturing costs, and complex processes persist [68, 237, 243, 244]. Therefore, the integration of IL-2 therapy with other treatments, such as chimeric antigen receptor (CAR) T-cell therapy (discussed below) and anti-PD-1 antibodies, presents an intriguing landscape for innovative therapeutic strategies. These combined treatments in preliminary trials reveal promising outcomes, potentially addressing standalone IL-2 therapy limitations [253, 256, 257]. Consequently, to navigate the therapeutic potential and drawbacks of IL-2 molecules, research is ongoing in areas such as designing IL-2 therapeutics with enhanced in vivo half-lives, specific IL-2 receptor conformation targeting to stimulate particular T cell subsets, and localized therapies [241]. This concerted effort could lead to the evolution of more precise and effective IL-2-based therapies.

Interestingly, another burgeoning area of research is IL-2 therapy’s integration with other targeted therapies, such as tyrosine kinase inhibitors (TKIs) [10, 258, 259]. A preclinical study demonstrated improved anti-tumor activity when IL-2 was combined with a TKI targeting the mesenchymal-epithelial transition factor (MET) receptor in a mouse model of RCC [260]. Clinical trials are now underway to assess the combination of IL-2 and TKIs in patients with metastatic RCC [261, 262]. While IL-2-based therapies present certain challenges, they remain a key frontier in immunotherapy. Unquestionably, continuous research and clinical trials are expected to expand their scope and improve their therapeutic efficacy and safety [241, 251]. Despite the complexities, IL-2 therapies promise to significantly contribute to managing a broader range of cancer types and autoimmune diseases in the future..

## Novel approaches and future perspectives

### Receptor-gated IL-2 delivery via anti-human IL-2 antibody for regulatory T-cell activation

Receptor-gated IL-2 delivery is a unique therapeutic strategy that uses anti-human IL-2 antibodies to activate Tregs. In this technique, IL-2R functions as the ‘gate’, and the anti-human IL-2 antibody serves as the ‘key’ to unlock this gate. This selective delivery system ensures that IL-2 is selectively delivered to Tregs, promoting their activation and immunomodulatory functions [263].

The potential of receptor-gated IL-2 delivery to mitigate the side effects associated with conventional IL-2 administration, such as the unintended activation of various immune cells, makes it a promising therapeutic technique [263]. Furthermore, it has potential applications in situations where immune tolerance is compromised, like autoimmune diseases or transplantation [264]. A study that warrants particular attention utilized a cell-based and dynamic IL-2R platform to identify a distinct anti-human IL-2 antibody known as UFKA-20 [265]. UFKA-20 enabled selective and efficient stimulation of CD4 + Treg cells within freshly isolated human T cells ex vivo and in animal models in vivo [265]. However, it is crucial to calibrate this technique carefully to avoid excessive immune suppression that could potentially render the host vulnerable to infections or malignancies [71, 266].

### Bispecific antibodies

Bispecific antibodies (bsAbs) represent a novel class of bioengineered molecules that can simultaneously engage two distinct antigenic epitopes. These uniquely dual-targeting agents demonstrate potential in revolutionizing cancer immunotherapy by concurrently interacting with IL-2R and tumor-associated antigens (TAAs), thereby bolstering T-cell mediated anti-tumor immunity [267–270]. Preclinical models have shown promising results with bsAbs enhancing the efficacy of receptor-gated IL-2 delivery and improving treatment outcomes in cancer [271–273]. For instance, in preclinical studies, a bsAb targeting PD-1 and LAG-3 exhibited enhanced T-cell activation and anti-tumor efficacy [272]. This dual-targeting approach of bsAbs provides a promising direction for cancer immunotherapy [274].

### Multi-specific antibodies

Multi-specific antibodies (msAbs) can engage multiple antigens simultaneously, making them a valuable tool in cancer immunotherapy [268]. By targeting both IL-2 and co-stimulatory receptors like CD28 and 4-1BB, msAbs can enhance T-cell activation and proliferation, thereby improving the effectiveness of receptor-mediated IL-2 delivery and reinforcing anti-tumor immunity [152, 275]. Recent preclinical studies have shown promise in this area, demonstrating that msAbs can enhance anti-tumor immunity and the efficacy of receptor-gated IL-2 delivery [276, 277].

### Fc receptor engineering

Fc receptor engineering involves modifications of the Fc region of anti-human IL-2 antibodies to augment antibody-dependent cell-mediated cytotoxicity (ADCC) and complement-dependent cytotoxicity (CDC). This can increase the efficacy of receptor-gated IL-2 delivery, potentially contributing to more effective therapeutic approaches in cancer management [278, 279]. Researchers continue investigating the potential of engineering the Fc region of anti-human IL-2 antibodies to improve ADCC and enhance receptor-gated IL-2 delivery [280–283].

### Site-specific conjugation

Site-specific conjugation is attaching therapeutic molecules, such as drugs or toxins, to specific sites on an antibody molecule to create an antibody-drug conjugate (ADC). This technique can improve the selectivity and potency of cancer therapeutics by targeting specific antigens on tumor cells [41]. Site-specific conjugation has been used to develop several FDA-approved ADCs, such as ado-trastuzumab emtansine (Kadcyla) and brentuximab vedotin (Adcetris), for the treatment of HER2-positive breast cancer and CD30-positive lymphoma, respectively [42, 43].This method of attaching drugs or other molecules to specific sites on antibodies can improve their pharmacokinetic properties and reduce off-target effects. Recent studies have shown that site-specific conjugation can enhance the efficacy of receptor-gated IL-2 delivery and improve treatment outcomes in preclinical cancer models [44, 45].

Moreover, by enhancing the precision of IL-2 delivery through site-specific ADC conjugation, treatment outcomes can be improved significantly, as demonstrated by another recent preclinical cancer model [45, 46]. The aforementioned study underscores the potential of ADCs in cancer treatment, discussing the strategies for targeted drug delivery and how this precision may enhance the therapeutic potential of drugs [46]. These findings support the hypothesis that site-specific ADC conjugation can enhance IL-2 delivery, thereby improving treatment outcomes in cancer therapy. Further research in this domain is warranted to confirm these promising initial results and to explore possible applications in clinical settings.

### CAR T cells and superkines

CAR T-cell therapy is a rapidly evolving therapeutic strategy that has been widely recognized for its role in harnessing the immune system to combat malignancies [90]. A prominent research interest lies in the modulation of CAR T-cell functions and their persistence in vivo through a combination therapy approach [284]. One such approach is integrating CAR T-cell therapy with IL-2 or IL-2R. This combined therapy has gained considerable attention in recent years due to its potential to enhance CAR T-cell performance and augment anti-tumor efficacy [285, 286]. In the context of CAR T-cell therapy, IL-2 can enhance the function of CAR T cells and improve their anti-tumor efficacy [287, 288]. Recent studies have shown that the co-administration of an anti-IL-2 antibody with CAR T cells improved CAR T cells’ persistence and function in a mouse glioblastoma model [289]. These advancements suggest that IL-2 or IL-2R-based therapies can impact the therapeutic success of CAR T-cell therapies.

In line with this, recent findings indicate that an orthogonal human IL-2 and IL-2Rβnsystem, termed Ortho-hIL-2, enhances CAR T cell expansion and antitumor activity in a murine model of leukemia [249]. This system not only boosts CAR T cells but also enhances their cytotoxicity and promotes their expansion, leading to leukemia regression [249]. Other orthogonal IL-2/IL-2R systems have been similarly found to enhance CAR T-cell therapy’s effectiveness, aiding in controlling CAR T-cell function, maximizing efficacy, and preventing acute graft-versus-host disease [290].

Given the growing body of evidence on IL-2/IL-2R’s role in enhancing CAR T-cell therapies, some researchers are exploring engineering CAR T cells to produce IL-2 within the tumor microenvironment [291]. This innovative approach could potentially improve the persistence and activation of CAR T cells, aiding in the clearance of bulky tumors [290, 291]. Further studies are necessary to validate this concept and determine its applicability in treating different cancer types.

However, it is important to consider that IL-2 therapies may also have limitations, such as promoting T cell exhaustion and influencing T cell differentiation [109, 290]. Therefore, fine-tuning the use of IL-2 in CAR T-cell therapies may be necessary to maximize benefits while minimizing adverse effects. On this note, targeted IL-2 variants have been shown to enhance CD8 + T-cell response, improve tumor control, and overcome resistance, suggesting that more personalized IL-2 therapies may be feasible [292–294].

Looking ahead, the integration of IL-2/IL-2R into CAR T-cell therapies holds substantial promise for cancer treatment. As research progresses, it will be important to continue exploring optimal combinations and doses, and develop strategies to mitigate potential side effects. This exciting frontier of cancer therapy stands to transform the treatment landscape for various types of cancer.

The field of cancer therapeutics has seen a paradigm shift with the introduction of superkines. Superkines are derived from diverse cytokine libraries and designed for enhanced biological potency, offering an optimistic avenue for cancer treatment [295]. A pivotal focus is the integration of IL-2 and IL-2R, which are promising therapeutic agents due to their critical role in immune cell function, cytotoxicity, and regulatory T cell expansion [109, 216]. However, designing IL-2 superkines has necessitated advanced computational methodologies to enhance affinity and stability [296]. An engineered IL-2 variant, MDNA109, was created to maximize antitumor effects while minimizing immune-related side effects [295]. This high-affinity, thermostable variant was shown to have an outward conformation that prearranges the IL-2Rβ binding site, optimizing its signaling properties [296]. Notably, the integration of IL-2 with its receptor, IL-2R, has been engineered for therapeutic proteins to bind more efficiently. This enhanced affinity for IL-2Rβois a key step in IL-2’s potency in immunotherapy [296]. IL-2’s interaction with IL-2R leads to signal cascades within the cell that promote proliferation and survival of effector T cells and NK cells, essential components in immune responses against tumors [109]. Further advancements include the development of fusion proteins and antibody complexes [109]. IL-2/anti-IL-2 antibody complexes, for instance, have been used to manipulate the immune system’s response to cancer by selectively expanding desired immune cell populations, such as CD4 regulatory T cells, and combating T cell exhaustion, a phenomenon in which T cells lose their functional capacities in chronic diseases like cancer [109]. Recent studies have demonstrated the potential for reshaping the TME using superkines, particularly with MDNA109. Delivered by an oncolytic adenovirus, MDNA109 shows superior anti-tumor responses in pancreatic cancer by enhancing immune cell activity and anti-tumor immune memory [297]. Interestingly, superkines like MDNA109 might be beneficial for treating immunologically “cold” tumors, known for their low mutation rates and limited immune cell infiltration [297]. Despite the promising prospects of superkines, certain issues need addressing. While high-dose IL-2 was previously used for melanoma and RCC treatment, its efficacy is questioned due to the rise of more targeted therapies [298]. Balancing the beneficial and detrimental effects of IL-2-based therapies continues to be a challenge in this field. Looking ahead, continuous research aims to optimize the potential of IL-2/IL-2R integration in superkines. Future perspectives include refining the therapeutic application of superkines through in silico affinity maturation and structure stabilization strategies, advancing clinical trials for cancer and autoimmune diseases, and targeting IL-2 to specific tissues for a more precise therapeutic approach [80, 296, 298]. The realization of these prospects may hold the key to transforming cancer therapeutics, making superkines a beacon of hope in the fight against this relentless disease.

### Current limitations and challenges of IL-2-based therapies

IL-2 is a key cytokine in the gamma (c) family, with critical roles in the TME and various therapeutic applications. Gamma (c) cytokines, including IL-4, IL-7, IL-9, IL-15, and IL-21, are vital for immune regulation, with shared use of IL-2Rs across these cytokines contributing to their functional overlap and redundancy [299]. This receptor sharing could be a double-edged sword in therapeutic utilization, as it allows for broad immunomodulatory effects but could potentially lead to unintended off-target effects. IL-2’s primary role in the TME is to drive the proliferation and activation of cytotoxic T cells, effectively aiding in the elimination of tumor cells [300]. Moreover, IL-2 has been widely utilized in immunotherapy for its ability to promote the expansion and function of Tregs, which are instrumental in maintaining immune homeostasis and preventing autoimmunity [23]. However, the pleiotropic nature of IL-2 can also contribute to adverse effects such as vascular leak syndrome, presenting a challenge to its therapeutic use [91]. Comparative studies of gamma (c) cytokines in immunotherapeutic settings have demonstrated distinctive advantages and limitations. Markley and Sadelain (2010) highlighted how different gamma (c) cytokines, despite sharing IL-2Rs, can preferentially promote the expansion of distinct immune cell subsets [301]. For instance, IL-7 and IL-15, unlike IL-2, primarily support memory T cell survival and proliferation, offering potential benefits in long-term tumor control. However, the broad receptor sharing across these cytokines might also lead to the activation of unwanted cell populations, potentially aggravating immune-related adverse events. While IL-2 and other gamma (c) cytokines play pivotal roles in the TME and hold substantial promise in cancer immunotherapy, the therapeutic exploitation of these cytokines requires a delicate balance of maximizing antitumor efficacy while minimizing off-target effects and toxicity.

IL-2 and IL-2R-based therapies have emerged as significant breakthroughs in cancer immunotherapy, their role rooted in IL-2’s crucial functions in T cell biology and immune regulation [302]. Demonstrating marked efficacy in conditions like metastatic melanoma, renal cell carcinoma, and synovial sarcoma, they have further been enhanced by the advent of new IL-2 formulations such as Alb-IL2 and IL2-Fc, providing improved clinical outcomes [91, 93, 303]. However, despite these promising advancements, IL-2-based therapies are not devoid of limitations. IL-2’s inherent toxicity, notably its association with VLS, can impose restrictions on its therapeutic applications [10]. Factors such as angiopoietin 2 and endothelial nitric oxide synthase have been implicated in IL-2-induced VLS [93]. The influence of Tregs also critically determines the effectiveness of IL-2-based therapies. While IL-2 augments T cell responses, it concurrently promotes Tregs expansion, which can counteract anti-tumor immune responses [10, 303]. This expansion of Tregs could therefore detrimentally affect the efficacy of IL-2-based cancer immunotherapies. Moreover, the intricate administration protocols and restricted applicability of IL-2 therapy contribute to its limitations, as evidenced in the treatment of mRCC [303]. Another significant factor influencing the success of these therapies is the individual tumor immune microenvironment. Variability in response rates among melanoma patients undergoing immunotherapy may be attributable to differences in their specific tumor immune microenvironments [91]. Efforts are underway to improve the safety and efficacy of IL-2 based therapies by altering the molecule itself or by changing the way it’s administered. (Supplementary Table 1 [291, 304–318] summarizes the novel approaches for cancer immunotherapy).

## Summary

IL-2 is a cytokine crucial for the activation and proliferation of immune cells, including T cells and natural killer cells. In the tumor microenvironment, IL-2 and its IL-2R have complex and sometimes opposing roles in tumor progression and immune response. While IL-2 can stimulate immune cells to attack tumors, it can also promote the expansion of regulatory T cells that suppress anti-tumor immunity. Hence, maintaining the balance between IL-2 and IL-2R signaling is critical for effective anti-tumor immunity. In addition, research has demonstrated that various cancer types exhibit different levels of IL-2 and IL-2R expression, which can impact their response to immunotherapy. For example, melanoma and renal cell carcinoma have been shown to have high levels of IL-2R expression, making them more responsive to IL-2-based immunotherapy.

Moreover, soluble IL-2R and IL-2 alpha have also been examined as potential biomarkers for cancer diagnosis, treatment, and prognosis. Engineered forms of IL-2 have been developed to improve its anti-tumor activity while reducing toxicity. For example, pegylated IL-2 has been demonstrated to possess a longer half-life and greater efficacy than native IL-2. Clinical trials have investigated the use of IL-2 alone or in combination with other agents, such as checkpoint inhibitors, in treating various types of cancer, including melanoma and renal cell carcinoma.

Although IL-2-based therapies have exhibited promise in some cancer types, the complex interplay between IL-2 and the tumor microenvironment necessitates further investigation. In addition, future research may focus on optimizing dosing and combination strategies to enhance the effectiveness of IL-2-based immunotherapy and identifying patient populations that are most likely to benefit from this approach. Overall, the study of IL-2 and its receptor in cancer holds excellent potential for developing new and effective treatments for a wide range of malignancies.

## Conclusion

In conclusion, the role of IL-2 and IL-2R within the tumor microenvironment remains a fascinating and vital area of exploration. Their multifaceted interplay—mediating both immune activation and regulation—has profound implications on the immune response to various cancers, suggesting that their targeted manipulation holds promise for improving cancer immunotherapy outcomes. Emerging evidence supports diverse IL-2 and IL-2R expression levels across different cancer types, potentially shaping their responses to immunotherapy. This heterogeneity underscores the need for a personalized approach to IL-2-based immunotherapy, with future strategies potentially requiring tailoring based on individual patient characteristics and tumor profiles. The development and optimization of engineered forms of IL-2, such as pegylated IL-2, mark an exciting advancement in this field. These novel forms promise to enhance therapeutic efficacy while curbing systemic toxicity.

Furthermore, exploring combination therapies—IL-2/IL-2R-targeted therapies coupled with traditional cancer treatments like chemotherapy, radiotherapy, or other immunotherapies like checkpoint inhibitors—could optimize cancer treatment efficacy. As we continue to investigate the role of IL-2 and IL-2R in tumorigenesis, there is also growing interest in their potential as diagnostic, prognostic, and monitoring biomarkers in cancer. Their utility in this regard could provide invaluable insights to inform therapeutic selection and timing. While substantial progress has been made, much remains to be understood about the intricate dance between IL-2 and the tumor microenvironment. Future research should strive to elucidate this complexity and translate these insights into more effective, safe, and patient-specific therapies. As we navigate this challenging yet promising landscape, the full therapeutic potential of IL-2 and IL-2R-targeted treatments in cancer immunotherapy comes into sharper focus. Through this ongoing scientific exploration, we may ultimately improve cancer patient outcomes and transform the future of cancer treatment.

## Electronic supplementary material

Below is the link to the electronic supplementary material.

**Supplementary Table.1**: A Comprehensive overview of IL-2 and IL-2R based novel approaches in cancer treatment

## Data Availability

All data generated or analyzed during this study are included in this published article and its additional information files.

## List of abbreviations

ADCC: Antibody-dependent cell-mediated cytotoxicity
ADC: Antibody-drug conjugate
AMG 592: An Engineered IL-2 Molecule
bsAbs: Bispecific antibodies
CAR: Chimeric Antigen Receptor
CDC: Complement-dependent cytotoxicity
CD122: Interleukin-2 receptor subunit beta
CD132: Interleukin-2 receptor subunit gamma
CD25: Interleukin-2 receptor subunit alpha
CD28: Cluster of Differentiation 28, a co-stimulatory protein
CD4: Cluster of Differentiation 4, a glycoprotein found on immune cells
CTL: Cytotoxic T Lymphocyte
CTLA-4: Cytotoxic T-Lymphocyte-Associated Protein 4
FDA: Food and Drug Administration
Fc: Fragment crystallizable region
HER2: Human epidermal growth factor receptor 2
IFN-γ: Interferon Gamma
IL-2: Interleukin-2
IL-2R: Interleukin-2 Receptor
IL-2Rα: Interleukin-2 Receptor Alpha
IL-2Rβ: Interleukin-2 Receptor Beta
IL-2Rγ: Interleukin-2 Receptor Gamma
IL-2/UFKA-20 complex: A complex formed between IL-2 and the anti-human IL-2 antibody UFKA-20
JAK: Janus Kinase
LAG-3: Lymphocyte-activation gene 3
mAbs: Monoclonal Antibodies
msAbs: Multi-specific antibodies
NARA1: Not an actual abbreviation, but a specific type of Superkine molecule
NK: Natural Killer (Cells)
NKTR-214: A type of Engineered IL-2 Molecule
PD-1: Programmed cell death protein 1
rIL-2: Recombinant Interleukin-2
STAT: Signal Transducer and Activator of Transcription
TAA: Tumor-associated antigen
TCR: T-Cell Receptor
TGF-β: Transforming Growth Factor Beta
TIL: Tumor Infiltrating Lymphocyte
TME: Tumor Microenvironment
Tregs: Regulatory T Cells
VEGF: Vascular Endothelial Growth Factor
VLS: Vascular Leak Syndrome
