# Supplementary material for: Reigniting hope in cancer treatment: the promise and pitfalls of IL-2 and IL-2R targeting strategies
Source: Mol Cancer. 2023 Jul 29;22:121. doi: 10.1186/s12943-023-01826-7 (PMC10385932; doi:10.1186/s12943-023-01826-7)
Supplement: Supplementary file 1 — Supplementary Table.1: A Comprehensive overview of IL-2 and IL-2R based novel approaches in cancer treatment [file 12943_2023_1826_MOESM1_ESM.docx]

| **Novel Approaches** | **Description** | **Type of Therapy** | **Examples** | **Mechanism of Action** | **Advantages** | **Disadvantages** | **Type of Cancer with Examples** | **Potential Applications** | **References** |
| --- | --- | --- | --- | --- | --- | --- | --- | --- | --- |
| IL-2 based Immunotherapy | Use of recombinant IL-2 to stimulate immune response against cancer cells | Immunotherapy | Aldesleukin, Proleukin | Stimulates the growth and differentiation of T cells, B cells, natural killer cells, and macrophages | Can stimulate potent immune response, Approved for renal cell carcinoma and melanoma | High doses can cause severe side effects like capillary leak syndrome | Renal cell carcinoma, Melanoma | Could be extended to other cancers with high immune cell infiltration | (6, 10, 81, 91) |
| CAR-T Cells Engineered to Secrete IL-2 | Genetically modified T cells to express CARs and secrete IL-2 | Cellular Therapy | Experimental stage | Enhances the persistence and efficacy of CAR-T cells, boosting immune response | Potentially more effective than conventional CAR-T therapy | Still in experimental stage, may have risks related to CAR-T cell therapy | Solid tumors, Leukemias, Lymphomas | Improving efficacy of CAR-T therapy | (256, 284, 289, 294, ) |
| IL-2/IL-2R targeted Antibody-Drug Conjugates (ADCs) | Antibodies targeting IL-2 or IL-2R conjugated with cytotoxic drugs | Targeted Therapy | Experimental stage | ADCs deliver the cytotoxic drug specifically to the cancer cells expressing IL-2 or IL-2R | Highly specific, reduces off-target effects | Potential for resistance, limited to cancers expressing IL-2 or IL-2R | Any cancer expressing IL-2 or IL-2R | Developing highly specific targeted therapies | (278, 279, 280-283) |
| IL-2/IL-2R Bispecific Antibodies | Antibodies designed to bind two different antigens, one of which is IL-2 or IL-2R | Targeted Therapy | Experimental stage | Directs immune cells to tumor cells expressing IL-2 or IL-2R | Can enhance specificity and efficacy of immune response | May cause off-target effects, still in experimental stages | Any cancer expressing IL-2 or IL-2R | Creating more effective targeted therapies | (267 -274) |
| IL-2 Fusion Proteins | Combination of IL-2 with other therapeutic proteins | Combination Therapy | Experimental stage | Enhances the range of therapeutic effects | Potential for synergistic effects | Could have increased side effects, still in experimental stages | Broad range of potential applications | Designing multifaceted therapies | (93, 109, 244, 291 304) |
| Nanoparticle Delivery of IL-2 | Using nanoparticles to deliver IL-2 to tumor cells | Drug Delivery | Experimental stage | Allows targeted delivery and controlled release of IL-2 | May improve efficacy and reduce side effects | Requires further development and testing | Potentially any solid tumor | Enhancing delivery of IL-2 therapies | (86, 305, 306) |
| IL-2 Cytokine Storm Mitigation | Development of therapeutics to manage IL-2-induced cytokine storm | Symptom Management | Experimental stage | Managing the inflammatory response triggered by high doses of IL-2 | Potential to make IL-2 therapy more tolerable | Not a direct treatment for cancer, still in experimental stages | Any cancer treated with high-dose IL-2 | Improving patient experience during IL-2 therapy | (81, 307 , 308) |
| IL-2 Mutant Proteins | Engineering IL-2 proteins with altered properties | Genetic Engineering | Experimental stage | Could enhance specificity or potency of IL-2 | Potential for increased efficacy or safety | Still in experimental stages | Potentially any cancer susceptible to IL-2 therapy | Tailoring IL-2 properties to specific therapeutic needs | (10, 93) |
| IL-2/IL-2R Antagonists | Compounds that block the activity of IL-2 or its receptors | Immunomodulatory | Experimental stage | Can inhibit Treg cells that suppress immune response | Could enhance anti-tumor immune response | Potential for off-target effects, still in experimental stages | Potentially any cancer with high Treg cell infiltration | Modulating immune response to improve immunotherapy | (309-311) |
| Inducible IL-2 Expression | Genetically modifying cells to express IL-2 under specific conditions | Gene Therapy | Experimental stage | Allows controlled expression of IL-2 | Could improve safety and efficacy | Still in experimental stages, potential for off-target effects | Potentially any cancer susceptible to IL-2 therapy | Tailoring IL-2 expression to specific therapeutic contexts | (3, 10, 312-314) |
| IL-2 Loaded Exosomes | Using exosomes to deliver IL-2 to tumor cells | Drug Delivery | Experimental stage | Allows targeted delivery and controlled release of IL-2 | May improve efficacy and reduce side effects | Requires further development and testing | Potentially any solid tumor | Enhancing delivery of IL-2 therapies | (315-318) |

**Supplementary Table.1**: A Comprehensive overview of IL-2 and IL-2R based novel approaches in cancer treatment
